# Occurrence and Composition of Microplastics in the Seabed Sediments of the Coral Communities in Proximity of a Metropolitan Area

**DOI:** 10.3390/ijerph15102270

**Published:** 2018-10-16

**Authors:** Chi Chiu Cheang, Yue Ma, Lincoln Fok

**Affiliations:** Department of Science and Environmental Studies, The Education University of Hong Kong, Tai Po, New Territories, Hong Kong 999077, China; cccheang@eduhk.hk (C.C.C.); s1110376@s.eduhk.hk (Y.M.)

**Keywords:** microplastic, sediments, coral, polymer, FTIR

## Abstract

In marine environments, microplastics have become a focus in scientific research in the last decade due to the global threat this pollutant poses to the marine environment. Corals in Hong Kong are under threat due to the degradation of the marine environment caused by human activities. This study investigated the occurrence, abundance and composition of microplastic debris (0.3–5 mm) in seabed sediments adjacent to coral communities in Hong Kong. Twenty-four benthic sediment samples were collected from four study sites located along the northeastern and eastern shores of Hong Kong. Microplastic concentrations ranged from 169 ± 48 to 221 ± 45 items/kg, and the mean concentration of microplastics in the seabed sediments was 189 ± 50 items/kg, which was comparable to similar studies in other regions. Microplastics accounted for 95.4% of particles extracted from benthic sediment samples using 40× light microscopy. ATR-FTIR spectroscopy analysis showed that polyethylene (PE) and polyethylene terephthalate (PET) comprised the majority of polymer types, contributing 45.3% and 29.3%, respectively. The proportion of microplastics made from PE and PET in seabed sediments was significantly higher than that observed in local beach sediments. The proportion of microplastics made from PE and low-density polyethylene (LDPE) and polypropylene (PP) together in the seabed sediments was much higher than that of PET and polyvinyl chloride (PVC). The results have provided information with reference to environmental concentrations of microplastics for fringe reef habitat close to urban areas, which can be applied in studies concerning ecotoxicity of microplastics.

## 1. Introduction

Plastic debris is a pervasive environmental issue, and the accumulation of plastic debris has been recognized as a global threat to marine organisms and seabirds. As more evidence on the occurrences and impacts of plastic debris is discovered in recent decades, it has become an important environmental concern [1,2]. Despite the increasing attention at the international level, plastic debris continues to accumulate in the environment, mainly due to its mass production and the leakage of disposed plastic products from waste management systems [3]. In 2010, 192 coastal countries generated approximately 275 million tonnes of plastic waste, which is equivalent to the global production of plastic materials [4]. In addition, it was estimated that up to 12.7 million tonnes of plastic waste entered the oceans each year [5].

In the marine environment, large plastic particles can be broken down into smaller fragments by wave action, hydrolysis and photodegradation [6,7,8]. When plastic debris is smaller than 5 mm in diameter, it is known as microplastic, which has been the target and focus of scientific studies due to the threat it poses to the marine environment [6]. In addition, cosmetics and fabrics can contain microplastic particles and can enter the marine environment since they are not completely retained by sewage treatment processes [9].

Microplastic pollution has been found in various environments. Small plastic, which was dominated by millimetre-sized items, was found floating on the sea surface at a relatively low concentration (1.1 g/km^2^) [10]. On beaches, where microplastics commonly accumulate, abundances ranged from 27 particles/m^2^ to 5595 particles/m^2^ [8,11]. A recent study also found that the low-energy mudflats have considerably high concentrations (from 0.58 to 2116 items/kg) of microplastics [12]. In the sea surface of continental shelfs, the concentration of microplastics ranges from 3 to 102,000 particles/m in the water body [13,14]. Alarmingly, microplastic particles have also been found to reach the most remote areas of marine environments, such as the deep sea. The abundance of microplastics was found to be from 0 to 400 particles/m for the sediments collected from depths between 1176 and 4844 m [3]. The ubiquitous nature of this issue has affected local recreational use of the coastlines and the quality of life in Hong Kong [15]. The ubiquitous presence of microplastics also means that the pollutant has a potential to affect a variety of organisms, including those intended for human consumption. For example, 83% of Norway lobsters had ingested microplastics [16]. Studies about the ingestion of microplastics by marine organisms in China are relatively scarce. Cheung, et al. [17] documented the ingestion of plastics in 60% of wild mullet samples. Another study found microplastics existed in tissue of all bivalves sampled from China’s markets, with four to 57 particles detected per individual [18]. In the Pearl River Estuary, plastics were also found in wild oysters, with one to seven particles detected per individual [19].

The coral reef ecosystem is regarded as one of the most sophisticated and highly productive marine ecosystems in the world and is believed to support a high diversity of marine organisms and provide ecosystem services to mankind [20]. Various chemical pollutants such as pesticide, trace metals and petroleum hydrocarbons were found in coral reef and affect different organisms in the system [21]. For example, herbicide glyphosate can act synergistically with elevated temperature and induce bleaching of the scleratinian (hard) corals [22]. In addition, Shaw, et al. [23] demonstrated the phytotoxicity of herbicides on coral-symbiotic zooxanthellae. Oxybenzone, an organic compound used in sunscreens, is another example which has adverse effects on corals and could lead to disruption of endocrine, damage to DNA, and deformed and irregular growth [24]. Although whether microplastics in the environment can significantly harm marine organisms is still under debate [25], microplastics can adsorb toxic chemicals, such as polychlorinated biphenyls (PCBs) and polycyclic aromatic hydrocarbons (PAHs) is a known fact. The concentration of hydrophobic pollutants on microplastics can be a million times higher than that observed in surrounding seawater [26]. More recently, Lohmann [27] suggested that microplastics themselves may be considered as persistent organic pollutants (POP).

A recent laboratorial study demonstrated that hard corals can consume microplastics [28]. Blue polypropylene (PP) particles of 10 μm to 2 mm with density of around 0.4 g/L were fed to the fragments of corals *Dipsastrea pallida*. Over 20% of the polyps studied had ingested at least one PP particle. Allen, et al. [29] showed that chemoreception played an important role in the consumption mechanism. Reichert, et al. [30] further found that the practices of microplastic consumption varied from species to species. Four types of responses were observed from the polyps that were exposed to microplastics (PE: polyethylene), namely attached particle on tentacles or mesenterial filaments, ingestion, mucus production and overgrowing on the particles. The species with large polyp sizes, such as *Acropora* spp. and *Pocillopora* spp., ingested the particles, whereas the species with small sizes like *Porites lutea* secreted mucus instead. Tissue necrosis and bleaching occurred in various species, but *P. lutea* remained healthy in the experiment [30].

Although there were studies in the laboratorial setting showing the potential impact of microplastic on corals, the implications of microplastic contamination in the coral reef area cannot be fully elucidated unless we could determine both the adverse physiological effect of microplastics on scleractinian coral and the relevant environmental concentrations of microplastics in the coral reef area. To bridge the knowledge gap regarding the latter issue, this study investigated the occurrence and composition of microplastics in seabed sediments of the coral communities in close proximity to a metropolitan area: Hong Kong.

## 2. Materials and Methods

### 2.1. Study Area and the Coral Communities

**Figure 1:**
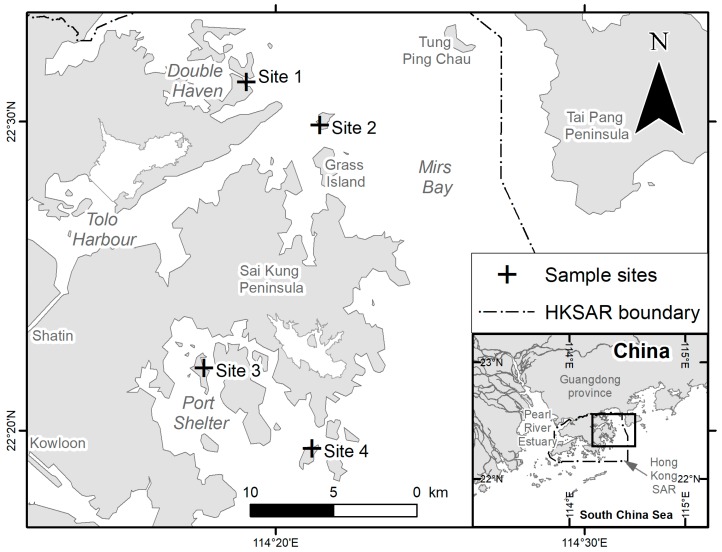
Map of four sample sites of this study. Site 1: Double Island; Site 2: Port Island; Site 3: Sharp Island; Site 4: Bluff Island.

Hong Kong is a megacity with a population of more than seven million people. It is located on the eastern shore of the Pearl River Estuary in China (Figure 1). The city itself as well as the Pearl River are the two major sources of microplastic pollution in Hong Kong [12,31]. Influenced by the freshwater discharge from the Pearl River, the waters in western Hong Kong are estuarine and turbid, rendering the area sub-optimal for coral growth. In contrast, marine waters along the eastern and northeastern shores are protected by natural shelters and are almost unaffected by discharge from the Pearl River. Therefore, corals in Hong Kong are primarily distributed in this region [32].

The coral communities in Hong Kong face various threats including storm damage [33], predation by corallivory gastropods [34] and sea urchins [35], and eutrophication [36]. The effect from microplastic or plastic pollution, however, has not yet been documented.

Based on the distribution of corals, two sites along the northeastern shore, i.e., Double Island (Site 1) and Port Island (Site 2), and another two sites along the eastern shore, i.e., Sharp Island (Site 3) and Bluff Island (Site 4), were selected (Figure 1). Abundant and diverse scleractinian corals, including *Acropora* spp., *Porites lutea* and other *Porites* species that were investigated in the feeding experiment of Reichert, et al. [30], were present at all of the sampling locations.

### 2.2. Sample Collection

Sediment samples were collected from the seabed by SCUBA divers in March and April 2017, although three out of the six samples from Site 4 were collected in August 2015. Nevertheless, the microplastic concentrations of the samples collected from Site 4 during the two time slots were not significantly different; thus, these samples were assumed to be temporally representative. Benthic sediment was collected at an average water depth of 4 m, which is the depth inhabited by scleractinian corals. At each site, six locations on the seabed were selected haphazardly, and these locations were adjacent to coral communities. Sediments were collected in accordance to the procedure adopted by Fok and Cheung [8] with minor modifications. At each location, sediments were collected from the top 4 cm of the seabed surface using a metal spade. The sediment was then transferred to a sealable and labelled plastic bottle. A total volume of 1 L of sediment sample was collected at each site. All samples were transported to the laboratory on the same day for storage and subsequent analysis.

### 2.3. Extraction Procedures

The extraction procedures were performed in accordance to Masura, et al. [37], albeit with minor modifications. Samples were successively wet-sieved through a 5-mm sieve and a 0.3-mm sieve to separate particles with diameters between 0.3 mm and 5 mm. Then, density separation was performed by adding aqueous zinc chloride (d = 1.6 g/cm^3^) solution to the samples. Finally, wet peroxide oxidation (WPO) was conducted to digest the organic materials. All solutions that would come into contact with the samples were filtered before use.

### 2.4. Identification of Microplastics

#### 2.4.1. Microscope Examination

Under a dissecting microscope (Olympus, Tokyo, Japan) at 40× magnification, the identifiable microplastics were collected and added to a watch glass using a pointed tweezer, according to the criteria set by Norén [14]: (1) absence of cellular or organic structures; (2) colours are homogeneous and distinct; (3) if it is a fibre, it is equally thick and does not taper to the ends. Additionally, it has a three-dimensional bending, instead of a completely straight shape. (4) For transparent or whitish pieces, extra examination should be carried out under a microscope with high magnification to rule out an organic origin. According to Cheung, et al. [31], the identifiable microplastics (diameter 0.3–5 mm) were sorted into five groups: (1) expanded polystyrene (EPS); (2) fibre (FB); (3) film (FL); (4) hard fragments (FM); and (5) pellets (PL).

#### 2.4.2. ATR-FTIR Analysis

The attenuated total reflectance—Fourier transform infrared spectroscopy (ATR-FTIR; PerkinElmer Frontier; Llantrisant, UK) technique was used to characterize the sorted microplastics based on polymer type. The ATR unit is equipped with a diamond top-plate. The spectra range between 4000 and 600 cm^−1^ was adopted for the FTIR analysis. The composition of plastic was characterized by comparing the sample spectra with standard spectra of the NICODOM polymers library, and this was performed using the spectrum application (version 10.03.07.0112; PerkinElmer, Waltham, MA, USA). A background scan was carried out prior to running each batch of samples. The spectrum of each sample was obtained by averaging four consecutive scans. Positive identifications were determined by a search score higher than 0.7. Twenty percent of the sorted microplastics were selected randomly for ATR-FTIR analysis. Samples were classified into 6 types according to their composition: (1) polyethylene (PE); (2) polyethylene terephthalate (PET); (3) polypropylene (PP); (4) polystyrene (PS); (5) polyvinyl chloride (PVC); and (6) mixed and other plastics (OTHERS). Out of the total of 380 instrumentally characterized microplastic items (21.1% of all samples), 362 (95.3%) were plastics of the above types.

### 2.5. Statistical Analysis

The abundance of the types of microplastic was presented using descriptive statistics. One-way analysis of variance (ANOVA) was used to test whether there were significant differences in microplastic abundance among the sample groups. Multivariate analysis was conducted using PRIMER-E [38] to reveal if there was any compositional difference among sites in terms of plastic types. The abundances of various types of microplastics were converted into a matrix (assemblage) by calculating the pairwise Euclidean distance among the samples (replicates of the sites). All sediment samples (24 samples) were grouped according to their sampling site. Non-metric multidimensional scaling (nMDS) multivariate analysis was carried out to visualize the compositional differences among the sediment samples, while ANOSIM was used to test if there was any significant compositional difference among the sites.

## 3. Results

A total of 1805 microplastic items were collected from 24 sediment samples from the four sites in this study. The mean concentration of microplastics in the sediments of the four sites ranged from 171.7 ± 57.6 to 223 ± 51.4 items/kg (±SD; Table 1). The overall mean concentration for the four sites was 194.5 ± 49.9 items/kg, which was nearly equal to the median value, i.e., 196.0 ± 39.5 items/kg (±MAD; Table 1). Among the 24 locations in the four sites, the concentrations of microplastics varied from 95 to 298 items/kg. The lowest and highest concentrations were recorded in Site 3 at Sharp Island and Site 2 at Port Island, respectively. The mean microplastic concentrations at the four sites did not exhibit a significant difference (ANOVA: *F* = 1.184, d*f* = 23, *p* = 0.341).

**Figure 2:**
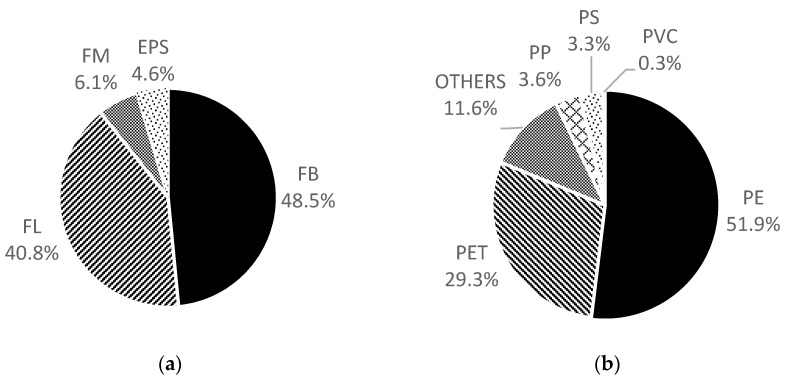
Proportion of microplastics by (**a**) type and (**b**) polymer.

Among the identifiable microplastic particles, fibres and films were the most commonly found, contributing to 48.5% and 40.8% of the total observed microplastics, respectively. Hard fragments and EPS accounted for the remaining 6.1% and 4.6%, respectively. No pellet was observed (Figure 2a).

**Figure 3:**
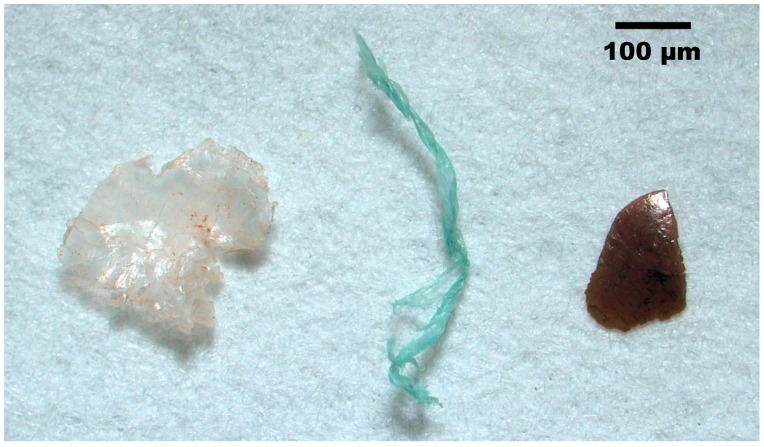
Micrograms of three types of microplastics. The scale bar indicates 100 μm. From left to right: film (FL), fibre (FB) and hard fragment (FM).

Micrograms of FB, FL and FM are given in Figure 3. A total of 362 characterized microplastic items were identified as plastics, and the accuracy of visual identification was 95.4%. Among these microplastics, most were polyethylene (PE; 51.9%) and polyethylene terephthalate (PET; 29.3%). The rest of the polymer groups constituted only a small proportion (Figure 2b). Multivariate analysis demonstrated that there is no significant grouping of samples with respect to their sites of collection (ANOSIM: R = −0.079; *p* = 0.885). Types of microplastics were not found to associate with any sample groups.

## 4. Discussion

The microplastic concentration (185 items/kg) observed in the coastal sediments of Hong Kong, as documented in this study, was compared to studies of similar nature (see Table 2).

The mean concentration is very similar to that observed for coastal sediments in Hong Kong and Yugoslavia, with concentrations of 158 items/kg and 177.8 items/kg, respectively. This comparison served as an indication of accuracy for the current survey, as these three studies adopted a similar particle size range for samples. The concentrations of microplastics observed in the study sites were only one-eighth of that observed in the Lagoon of Venice in Italy (1445 items/kg). When compared with beach sediment, the mean concentration of microplastics in the costal sediments of Hong Kong is approximately two orders of magnitude higher than that observed in Norderney of Germany (1.8 items/kg) and Singapore (2.3 items/kg). However, it is only approximately 3% of the mean concentration in some regions of China (6923 items/kg). It was not easy to directly compare the microplastic concentrations in different regions because the adopted sample size ranges were not unified among studies. Some researchers collected microplastics with diameters less than 5 mm, while others took samples of less than 1 mm. This difference could lead to an obvious variability of the results. In addition, the sampling methods, extraction methods and identification methods varied in the different studies, which could contribute to variation in the results as well. The unification and standardization of these sizes and methods regarding microplastics is needed to allow for comparison among studies. Furthermore, the presence of microplastics has also been observed on shorelines of an atoll in the Maldives [39].

Two explanations can possibly support this result. First, human activities are frequent in these areas, especially for Sharp Island and Bluff Island. Fishing is the primary means of how local fishermen make their living, and a large amount of derelict fishing gear can be found on the seabed of these areas. On a coastal clean-up day held at Sharp Island, 500 kg of waste was collected from the seabed and beach, and derelict fishing gear was the major component of the rubbish collected from the seabed [48]. Fishing nets are the main type of fishing gear used locally, and nets are made of PE and PET fibre [43]. This fact is consistent with the results of the microscope examination that found that fibres comprised the second largest share of the total detected microplastics; additionally, ATR-FTIR analysis demonstrated that PE and PET accounted for three quarters of the microplastics based on polymer types. In addition to fishing, sightseeing is a frequent human activity in these areas. Sites 3 and 4 are located in Sai Kung, and these are also popular diving sites for viewing corals. Visitors of these sites may discard plastics randomly or accidentally, and these plastics can include plastic bags and plastic water bottles. Compared with plastic bottles and other hard plastics, plastic bags are generally made of PE, and are softer and thinner. This means that they can be torn into pieces more easily; and thus, are more susceptible to be broken down into microplastics through wave action, hydrolysis and photodegradation [49]. Hence, the thin and soft microplastic debris coming from discarded plastic bags should be a key component of microplastic particles in the seabed at these sites. This fact is consistent with the results that showed that film and PE dominated the microplastics in terms of type and polymer, respectively.

Another explanation for the aforementioned result primarily concerns Sites 1 and 2, i.e., Double Island and Port Island, respectively. These two sites are located in Mirs Bay (also known as Dapeng Bay locally; Figure 1), which is close to Shenzhen City in Guangdong Province of China. The amount of microplastic debris present along the coastline of the South China Sea is massive, especially when compared with other areas [43,50]. Therefore, the coastline of Guangdong Province is a hotspot of microplastic pollution. Plastic particles could enter the marine environment by means of sewage discharge and illegal dumping in Mirs Bay [51], and it is known that approximately 80% of plastic particles in the marine environment originates from land-based sources [52]. The types of plastics from land-based sources are plastic bags and plastic bottles. PE and PET should be the dominant polymer types of the microplastics in Mirs Bay. This result agrees with that of Zhao, et al. [50], who found that PE (31.9%) was one of the most common polymer composition types in the South China Sea.

It is recommended that the beaches, sea surface, water column, and seabed should be monitored for marine debris [53]. In the study by Fok and Cheung [8], Hong Kong was identified as a hotspot of microplastic pollution by analysing sediments from 25 local sandy beaches. Among the microplastic items collected from the beaches, expanded polystyrene (EPS) made up the majority (92%). However, in this study, the majority of microplastics were PE and PET. Nonetheless, the level of microplastic pollution observed in seabed sediment was not as high as that observed on the beaches of Hong Kong. This difference may be attributed to the different rates of plastic debris degradation in the two environments. Plastic debris on the seabed sediments can maintain its size for a much longer time than can that in the beach sediments. This difference is because the breakdown rate on beaches is accelerated by the action of ultraviolet (UV) radiation, temperature, waves and wind [52]. UV is absorbed and scattered by seawater; thus, UV radiation rarely reaches the seabed sediments, and the temperature is much lower than that at the beach. In contrast, plastics sitting on beaches can absorb much more UV light, which is the catalyst of photodegradation. Beach surveys in the study by Fok and Cheung [8] were carried out between July and September 2014, i.e., in the summer. Hong Kong is situated in the south of the Tropic of Cancer, where summer is hot with high UV radiation on sunny days, and both of these characteristics can accelerate the breakdown of plastics in beach sediments. Given a steady input of large debris to the environment, these conditions give rise to a higher abundance of microplastics. Nevertheless, the same does not occur on the seabed, where plastic debris breakdowns slowly. As a result, the concentration of microplastics found on the seabed sediment is lower.

Differences in microplastic composition between beach and seabed sediments can also contribute to the differences in microplastic concentrations at these locations. The differences in polymer composition are partly due to the concentrations of the microplastics themselves. On the sea floor, less EPS and more PET was observed. The former has a very low concentration (below 0.05 g/cm^3^), while the latter (d ≈ 1.4 g/cm^3^) is denser than sea water (d = 1.02–1.03 g/cm^3^). The denser PET is negatively buoyant and sinks to the seabed quickly, which represents a stable source pathway. Moreover, the method of microplastic extraction was more conservative in the study by Fok and Cheung [8]. The medium used for density separation was seawater, and this method may not separate heavier plastics such as PET from beach sediments.

In addition, the heat-deformation temperature of both EPS and PET is relatively low (60–85 °C) compared to that of high-density polyethylene (90–110 °C), polypropylene (100–140 °C) and other plastics. Hence, the relatively high temperatures on beaches allow the most dominant form of microplastic, EPS, to breakdown, and this results in a high abundance of this type of microplastic on beaches.

In the study of Lo, et al. [12], microplastics from 10 mudflats spanning across the east coast and west coast of Hong Kong were quantified and identified. The mean abundance of microplastics in mudflat sediments was 268 items/kg. Among the total collected microplastic items, PE accounted for the majority of polymer types (46.9%), followed by PP (13.8%) and PET (13.5%). The average concentration (185 item/kg) of microplastics found in the current study was similar to that observed by Lo, et al. Likewise, PE represents the majority of microplastics in this study (51.9%), and the proportional abundance is very similar to that observed in Lo’s study. Some of the sample sites of Lo are in close proximity to those used in this study, and the samples in this study were collected in 2015 and 2017, while Lo, et al. collected their samples in 2016. In addition, the microplastic extraction methods used by the two studies were very similar. Both of these two studies used zinc chloride (d = 1.6–1.7 g/cm^3^) to separate microplastics from sediment. As aforementioned, almost all types of microplastics, especially for higher density microplastics, such as PET and PVC, float on zinc chloride solution. These characteristics all contributed to the similar results found in these two studies.

The majority of the microplastics found in this study are PE and PET, which had not been tested in the feeding experiments by Hall, et al. [28]. However, PE was indeed the testing microplastic particles in Reichert, et al. [30]. *Porites lutea* tested by Reichert also occurs in the sites of the current investigation. While Reichert at al. [30] found that *P. lutea* would secrete mucus but not ingest PE particles, those species of small polyps size found in the sites may share similar response of *P. lutea*, and do not consume microplastic directly. Whether the species of large polyps size in Hong Kong, such as *Acropora* spp. and *Platygyra* spp. (the most common local species), would consume microplastic in situ remains unclear. And further study is needed to extrapolate the results of feeding experiments to the field.

## 5. Conclusions

In this study, 24 samples from four sampling sites were collected and analysed. This is the first study to reveal the occurrence of microplastics in seabed sediments adjacent to the coral communities of the Hong Kong coastal zone. The results of this study show that the concentration of microplastics in sediments is comparable to that observed in other locations. In comparison with a local beach sediment study, the discrepancy of microplastic composition between beach sediments and seabed sediments in Hong Kong was demonstrated, which indicates a potential difference in the sources of microplastics. Nonetheless, since no coral has been sampled, it is uncertain whether microplastics or the associated organic contaminants, such as oxybenzone, have direct detrimental effects on corals. As a result, a toxicological study will be carried out to corroborate the adverse effects of microplastics on coral communities.

## Figures and Tables

**Table 1:** Descriptive statistics of microplastic concentrations (items/kg) observed for the four study sites. Site 1: Double Island; Site 2: Port Island; Site 3: Sharp Island; Site 4: Bluff Island.

Site	No. of Samples	Mean	Median	SD	CV%	MAD
1	6	185.0	194.5	38.3	20.7	25.0
2	6	171.7	168.5	57.6	33.6	51.0
3	6	223.0	221.0	51.4	23.1	38.5
4	6	198.5	198.5	47.9	24.1	42.0
All	24	194.5	196.0	49.9	25.7	39.5

**Table 2:** Concentrations of microplastics in marine sediments of different regions. The units of the microplastic concentrations have been standardized to items/kg.

Region	Site	Habitat	Particle Size (mm)	Concentration (items/kg)	Reference
Hong Kong, China	Double Haven, Tolo Harbour & Port Shelter	Coastal sea with fringing reef	0.3–5	Range: 95–298 Mean: 185	This study
Hong Kong, China	Deep Bay, Tolo Harbour, Tsing Yi & Victoria Harbour	Coastal sea	0.01–5	Range 44–458 Mean: 158	[40]
Belgium	Nieuwpoort, Oostende and Zeebrugge	Coastal sea	0.038–1	Maximum: 390 Mean: 166.7 ± 92.1	[41]
Italy	Lagoon of Venice	Lagoon	<1	Range: 672–2175 Mean: 1445	[42]
Hong Kong, China	Local coastal shores	Beach	0.25–5	Range: 0.58–2116 Mean: 161	[12]
China	Shapawan, Haikou, Wanning, Sanya and Beihai	Beach	<5	Range: 5014–8714 Mean: 6923	[43]
Germany	The Island of Norderney	Beach	<1	Range: 1–4 Mean: 1.8	[44]
Canada	Halifax Harbour	Beach	<5	Range: 2000–8000 (fibre)	[45]
Yugoslavia	Along the Slovenian coast	Beach	0.25–5	Maximum: 444.4 Mean: 177.8	[46]
Singapore	Local coastal shores	Beach	>0.0016	Maximum: 10.7 Mean: 2.3	[47]
